# Supplementary material for: Allosteric activation or inhibition of PI3Kγ mediated through conformational changes in the p110γ helical domain
Source: bioRxiv. 2023 Apr 12:2023.04.12.536585. Preprint. [Version 1] doi: 10.1101/2023.04.12.536585 (PMC10120615; doi:10.1101/2023.04.12.536585)
Supplement: Supplementary file 1 [file NIHPP2023.04.12.536585v1-supplement-1.pdf]

bioRxiv preprint doi: https://doi.org/10.1101/2023.04.12.536585; this version posted April 12, 2023. The copyright holder for this preprint (which was not certified by peer review) is the author/funder, who has granted bioRxiv a license to display the preprint in perpetuity. It is made available under aCC-BY 4.0 International license.

Supplemental information for:

Allosteric activation or inhibition of PI3Kγ mediated through conformational changes in the p110γ helical domain

Noah J Harris[1*], Meredith L Jenkins[1*], Sung-Eun Nam, Manoj K Rathinaswamy[1], Matthew Parson[1], Harish Ranga-Prasad[1], Udit Dalwadi[2], Brandon E Moeller[1], Eleanor Sheekey[1], Scott D Hansen[3], Calvin K Yip[2%], and John E Burke[1,2%]

[1]Department of Biochemistry and Microbiology, University of Victoria, Victoria, British Columbia, V8W 2Y2, Canada

[2]Department of Biochemistry and Molecular Biology, The University of British Columbia, Vancouver, British Columbia V6T 1Z3, Canada

[3]Institute of Molecular Biology, University of Oregon, Eugene, OR 97403

*These authors contributed equally

[%]To whom correspondence should be addressed: John E. Burke

Tel: 1-250-721-8732, email: jeburke@uvic.ca

Calvin K Yip

Tel: 1-604-827-3976, em ail: calvin.yip@ubc.ca

bioRxiv preprint doi: https://doi.org/10.1101/2023.04.12.536585; this version posted April 12, 2023. The copyright holder for this preprint (which was not certified by peer review) is the author/funder, who has granted bioRxiv a license to display the preprint in perpetuity. It is made available under aCC-BY 4.0 International license.

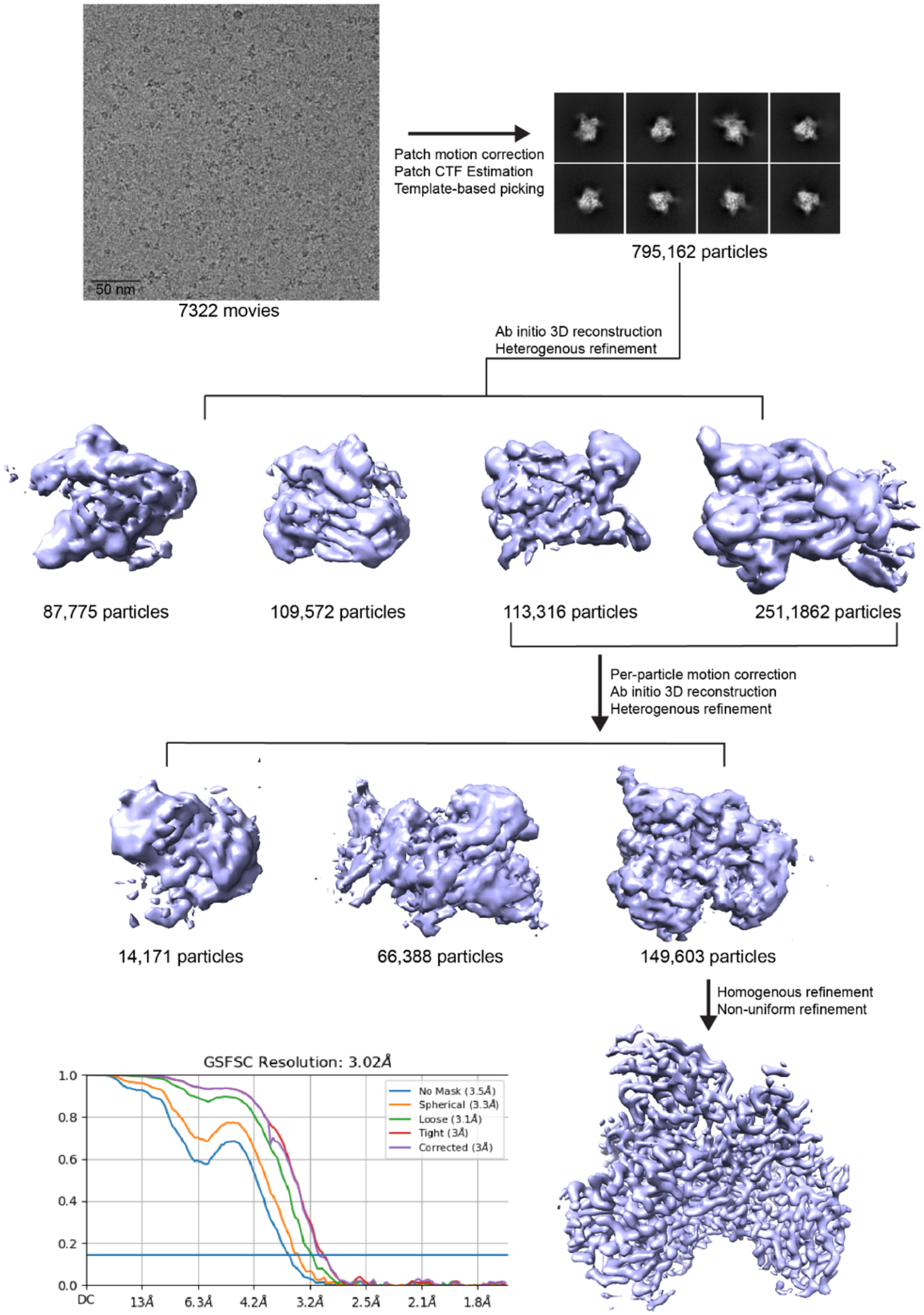

Figure S1. p110γ-NB7 complex cryo-EM analysis workflow (related to main figure 2): cryo-EM processing workflow of p110γ-NB7 complex are shown in order of a representative micrographs, representative 2D classification and 3D reconstruction processing strategy. Bottom left shows Gold-standard Fourier shell Correlation (FSC) curve of final round on non-uniform homogenous refinement.

bioRxiv preprint doi: https://doi.org/10.1101/2023.04.12.536585; this version posted April 12, 2023. The copyright holder for this preprint (which was not certified by peer review) is the author/funder, who has granted bioRxiv a license to display the preprint in perpetuity. It is made available under aCC-BY 4.0 International license.

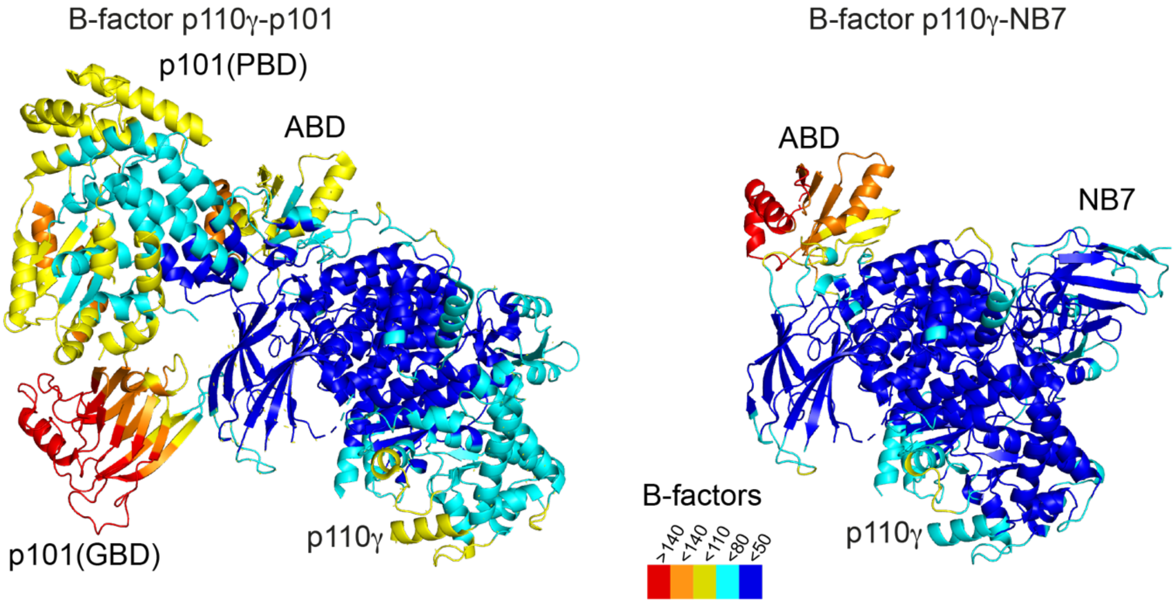

1169

1170 **Figure S2. Comparison of full length p110γ bound to NB7 compared to p110γ-p101 (related to**
1171 **main figure 2):** The structure of the p110γ-p101 complex (PDB:7MEZ) compared to the NB7-p110γ
1172 complex is shown colored according to B factor based on the legend.

bioRxiv preprint doi: https://doi.org/10.1101/2023.04.12.536585; this version posted April 12, 2023. The copyright holder for this preprint (which was not certified by peer review) is the author/funder, who has granted bioRxiv a license to display the preprint in perpetuity. It is made available under aCC-BY 4.0 International license.

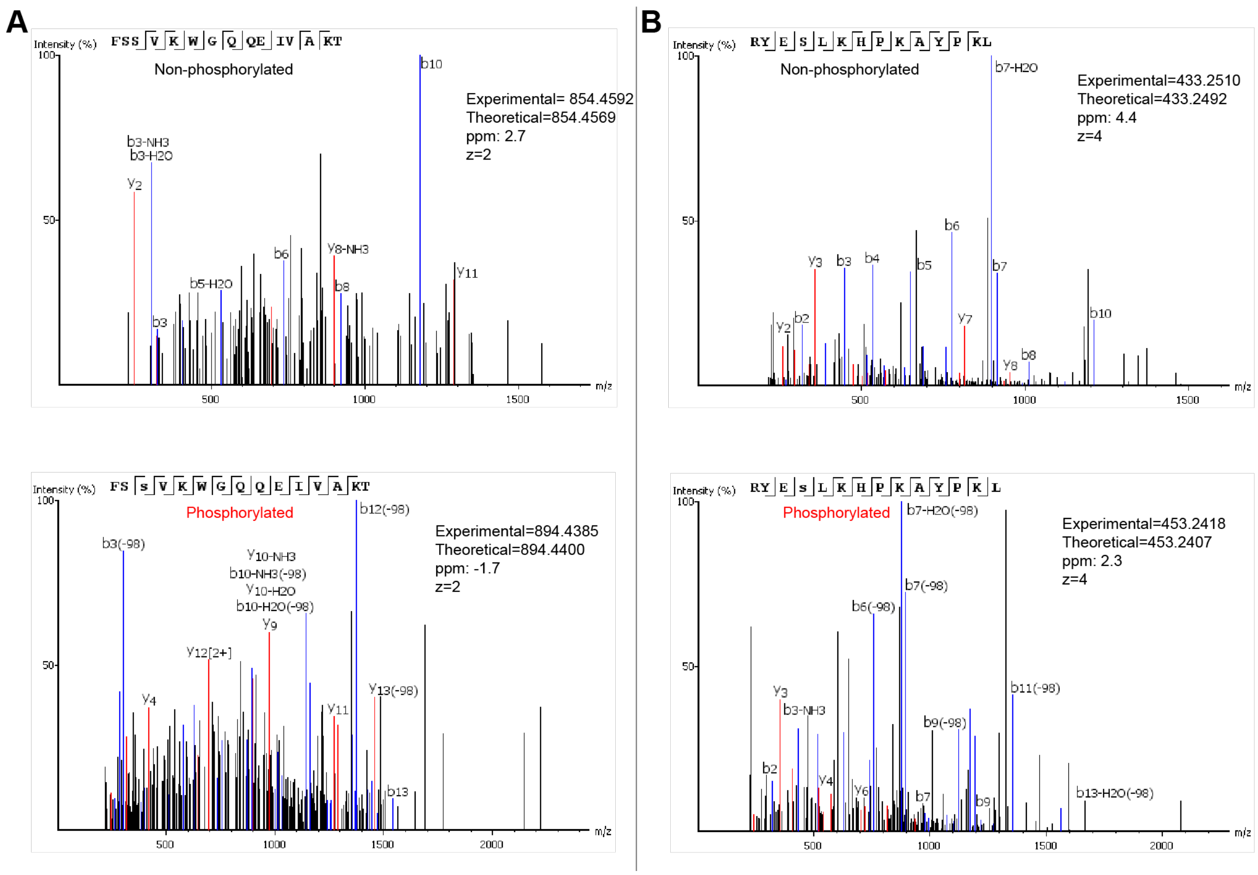

Figure S3 (related to main figure 3). MS/MS spectra of peptides spanning S582 and S594/S595 for both phosphorylated and unphosphorylated states. The theoretical and experimental mass are annotated for all peptides.

bioRxiv preprint doi: https://doi.org/10.1101/2023.04.12.536585; this version posted April 12, 2023. The copyright holder for this preprint (which was not certified by peer review) is the author/funder, who has granted bioRxiv a license to display the preprint in perpetuity. It is made available under aCC-BY 4.0 International license.

Supplementary table 1. Cryo-EM data collection, refinement and validation statistics **(related to main figure 2)**

	p110γ-NB7<br>EMD- 27627<br>PDB: 8DP0
**Data collection and processing**	
Magnification	
Voltage (kV)	300
Electron exposure (e/ Å^2)	50
Defocus range (nM)	500-2500
Pixel size (Å)	
Symmetry imposed	C1
Initial particle images (no.)	795,162
Final particle images (no.)	149,603
Map resolution (Å)	3.02
FSC threshold	0.143
Map resolution range (Å)	2.6-4.4
**Refinement**	
Initial model used (PDB)	7MEZ (p110γ only)
Model Resolution (Å)	3.02
FSC threshold	0.5
Map sharpening B factor	Sharpened locally
Model composition	
Non-hydrogen atoms	8737
Protein residues	1,066
Ligands	0
B-factors	
Protein	52.4
Validation	
Mol probability score	1.29
Clashscore	5.33
Poor rotamers (%)	0.0
Ramachandran	
Favored	98.41
Allowed	1.59
Outliers	0.0
R.m.s. deviations	
Bond lengths (Å)	0.002
Bond angles (°)	0.490
Model to map fit (CC_mask)	0.86

bioRxiv preprint doi: https://doi.org/10.1101/2023.04.12.536585; this version posted April 12, 2023. The copyright holder for this preprint (which was not certified by peer review) is the author/funder, who has granted bioRxiv a license to display the preprint in perpetuity. It is made available under aCC-BY 4.0 International license.

Supplementary table 2. HDX-MS data collection and validation statistics **(related to main figure 4)**

Data set	p110γ unphosphorylated	p110γ phosphorylated
HDX reaction details	%D_2O=75.5%<br>$pH_{(read)}$=7.5<br>Temp=4°C, 20°C	%D_2O=75.5%<br>pH(read)=7.5<br>Temp=4°C, 20°C
HDX time course (seconds)	3s at 4°C, 3s, 30s, 300s, 3000s at 20 °C	3s at 4°C, 3s, 30s, 300s, 3000s at 20 °C
HDX controls	N/A	N/A
Back-exchange	No correction, deuterium levels are relative	No correction, deuterium levels are relative
Number of peptides	244	244
Sequence coverage	98.4%	98.4%
Average peptide /redundancy	Length= 15.2<br>Redundancy= 3.3	Length= 15.2<br>Redundancy= 3.3
Replicates	3	3
Repeatability	Average StDev=0.53%	Average StDev=0.57%
Significant differences in HDX	>5% and >0.4 Da and unpaired t-test ≤0.01	>5% and >0.4 Da and unpaired t-test ≤0.01
